# Magnetic resonance imaging patterns of tumor response to chemotherapy in desmoid‐type fibromatosis

**DOI:** 10.1002/cam4.3973

**Published:** 2021-06-08

**Authors:** Edoardo Zanchetta, Chiara Maura Ciniselli, Annalisa Bardelli, Chiara Colombo, Silvia Stacchiotti, Giacomo Giulio Baldi, Salvatore Provenzano, Rossella Bertulli, Federica Bini, Alessandra Casale, Francesca Gabriella Greco, Andrea Ferrari, Paolo Verderio, Marco Fiore, Alessandro Gronchi, Paolo Giovanni Casali, Carlo Morosi, Elena Palassini

**Affiliations:** ^1^ Postgraduation School in Radiodiagnostics Università degli Studi di Milano Milan Italy; ^2^ Unit of Radiology ASST Santi Paolo e Carlo Milan Italy; ^3^ Diagnostic and Interventional Radiology Fondazione IRCCS Istituto Nazionale dei Tumori Milan Italy; ^4^ Unit of Bioinformatics and Biostatistics Department of Applied Research and Technological Development Fondazione IRCCS Istituto Nazionale dei Tumori Milan Italy; ^5^ Sarcoma Service Department of Surgery Fondazione IRCCS Istituto Nazionale dei Tumori Milan Italy; ^6^ Medical Oncology Unit 2 Medical Oncology Department Fondazione IRCCS Istituto Nazionale dei Tumori Milan Italy; ^7^ Oncological Pediatrics Fondazione IRCCS Istituto Nazionale dei Tumori Milan Italy; ^8^ Department of Oncology and Haemato‐Oncology Università degli Studi Milano Italy

**Keywords:** aggressive fibromatosis, magnetic resonance imaging, methotrexate, tumor response, vinca alkaloids

## Abstract

**Background:**

We aimed to investigate changes in volume and MRI T2‐weighted intensity in desmoid‐type fibromatosis (DF) receiving methotrexate plus vinca‐alkaloids (MTX‐VA) at Istituto Nazionale dei Tumori, Milan.

**Methods:**

All cases of sporadic DF treated with MTX‐VA from 1999 to 2019 were reviewed. MRIs at baseline, 6 and 12 months of chemotherapy and at treatment withdrawal were retrospectively reviewed, contouring the tumor lesion and measuring diameters, volume, and mean T2‐signal intensity (normalized to muscle) changes. These parameters were also evaluated according to clinical variables.

**Results:**

Thirty‐two DF patients were identified. Best RECIST response was: 25% partial response, 69% stable disease, 6% progression. A ≥65% tumor volume reduction was observed in 38%, <65% reduction in 53%, an increase in 9%. 22% had RECIST stable disease with a ≥65% tumor volume reduction. T2‐signal intensity decreased by ≥50% in 47%, <50% in 41% and increased in 12%. In patients with symptomatic improvement while on therapy and in patients maintaining symptomatic improvement during follow‐up, median T2‐signal intensity showed a reduction along the time points (3.0, 1.9, 1.2, 1.1; 2.9, 2.0, 1.2, 1.2, respectively); in patients without symptomatic improvement and in those clinically progressing during follow‐up, a reduction was not observed. High T2‐signal intensity at baseline was observed in patients showing RECIST progression during follow‐up.

**Conclusions:**

In this series, RECIST detected a lower proportion of responses as compared to volumetric and T2‐signal changes. T2‐signal reduction seemed to better reflect symptomatic improvement. High T2‐signal intensity at baseline was related to a higher proportion of further progression.

## Graphical abstract

In this series of patients with sporadic desmoid‐type fibromatosis treated with low‐dose chemotherapy and evaluated with MRI, T2 signal reduction was detected in more patients as compared to RECIST responses and volume reductions, and seemed to better reflect symptomatic improvement and worsening. RECIST may have underestimated treatment responses as compared to volumetric and T2 signal intensity changes. A high T2 signal intensity at baseline seemed to be related to a higher risk of RECIST progression after the end of chemotherapy.

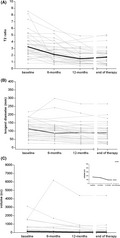

## 1 INTRODUCTION

Desmoid‐type fibromatosis (DF), also referred to as aggressive fibromatosis or desmoid tumors, represents a rare fibroblastic proliferative disease, with an incidence of 2–4 per million per year.^1^ Generally sporadic, in about 5–10% of cases it occurs in association with familial adenomatous polyposis (FAP) in Gardner's Syndrome. DF has a female predominance, with a peak incidence in subjects aged between 25 and 35 years. Extremities and girdles, together with abdominal wall, are the most frequent sites in the sporadic setting. In this setting, the intra‐abdominal site is rare, but it represents the main primary site in FAP patients.^2^ Although lacking metastasizing potential, DF is characterized by a local aggressiveness that, depending on the site, may result in functional impairment and/or pain. Moreover, stabilizations and regressions were reported in absence of any active treatments.^3^, ^4^

Given the high rate of local recurrence after surgery and, conversely, the possible indolent behavior of the disease, the role of surgery, previously viewed as the standard treatment, has been challenged and indeed is now felt to be much less important.^5^, ^6^, ^7^ In the last few years, European experts agreed upon an approach based on active surveillance, at least in asymptomatic patients^8^ : a systemic therapy is offered to patients becoming symptomatic or showing disease progression. A recent global consensus confirmed this approach.^9^ Several systemic therapies have shown to be active in DF, including anti‐estrogens^10^ and non‐steroidal anti‐inflammatory drugs, low‐dose chemotherapy with methotrexate plus vinca‐alkaloids (MTX‐VA),^11^ anthracycline‐based chemotherapy, and tyrosine‐kinase inhibitors.^12^, ^13^, ^14^, ^15^

In the management of any systemic therapy, a major role is played by tumor response assessment through imaging.^16^ As in other tumors, also in DF, the employment of one‐dimensional criteria to evaluate treatment effect, such as the currently used Response Evaluation Criteria In Solid Tumors (RECIST), is debated. Some authors have suggested that a decrease in MRI T2‐signal intensity may represent an indicator of treatment response in this disease.^14^ T2‐signal intensity reflects water content within tissues^17^ and can therefore be considered a surrogate for cellular content (in contrast to fibrous, extracellular matrix components). Of course, in this chronic, non‐malignant disease, that may cause functional impairment and pain, with a significant impact on quality of life, it is crucial to assess tumor response also through symptom assessment.

In this view, we made a retrospective study aiming to investigate changes in volume and T2‐weighted MR signal intensity and symptomatic changes in sporadic DF treated with low‐dose MTX‐VA at Fondazione IRCCS Istituto Nazionale dei Tumori, Milan, Italy (INT).

## 2 MATERIALS AND METHODS

All cases of sporadic DF treated at INT, from May 1999 to June 2019, with low‐dose weekly MTX‐VA and evaluated by MRI were retrospectively reviewed. FAP‐related DF was excluded. Cases were retrieved from a prospectively maintained database including all the DF patients treated at INT. Institutional ethics committee's approval was obtained. Patient medical records were examined to collect the following data: age, gender, tumor site, prior therapies, symptoms at baseline, chemotherapeutic regimen, reason for therapy discontinuation, symptomatic changes during chemotherapy and after therapy withdrawal, and additional treatments during follow‐up. MTX‐VA was administered weekly until reaching a number of cycles between 40 and 50, or until reaching one year of treatment, or the evidence of progression or toxicity.^11^

MRI at baseline, at 6 and 12 months of chemotherapy, at the end of treatment, and yearly during follow‐up were retrospectively extracted from the institutional Picture Archiving and Communication System and analyzed. Follow‐up data were censored at the date of starting a further treatment or at the last patient contact, in case of event‐free patients. Patients evaluated by CT or whose MRI examinations were not available or did not include T2‐weighted sequences were excluded. A radiologist performed manual segmentation of each entire tumor lesion in a slice‐by‐slice fashion using *IntelliSpace Tumor Tracking* software (*Philips*); three orthogonal diameters, volume, and mean T2‐signal intensity of each lesion (which was then normalized to the muscle intensity) were obtained from all the available MRI examinations.

For each of the three pivotal variables (longest diameter, normalized T2‐signal intensity, and volume), descriptive statistics were used to describe the radiological data at each time point (baseline, 6‐months, 12‐months, end of therapy) by considering their original continuous scale. Spaghetti plots were used to graphically depict the patient's trends over time, as well as their median trends. Moreover, the same analysis and graphical representation were performed by considering as pivotal variables the relative change from baseline (i.e. percentage of baseline). Then, for each radiological parameter, the best response during treatment was defined as the maximum reduction observed among the considered time points relative to the baseline values. A bar plot was adopted to graphically depict these patterns among patients. In addition, to aid the interpretation of the results, each pivotal variable was evaluated according to pre‐specified cut‐off values. Specifically, one‐dimensional response was categorized according to RECIST 1.1^18^ ; for the reduction of tumor volume, a 65% threshold was used to categorize response, since this corresponds to a 30% reduction in maximum tumor diameter, as per RECIST^19^ ; for T2‐signal intensity, a 50% reduction threshold was arbitrarily chosen as a cut‐off for response. Moreover, to grasp as much as possible information from the data, spaghetti plots were used to graphically depict the patient's trends over time according to four “clinical” variables: (1) symptomatic improvement or lack of improvement during therapy in patients reporting symptoms at baseline. Symptomatic improvement was defined as the improvement of at least one symptom, without the onset of new symptoms or worsening of other symptoms. Lack of improvement was defined as persistence or worsening of symptoms and/or onset of new symptoms; (2) clinical progression or lack of clinical progression after chemotherapy withdrawal. Clinical progression was defined as worsening of at least one symptom and/or onset of new symptoms. Lack of clinical progression was defined as the absence of both worsening of symptoms and onset of new symptoms; (3) Additional treatments after the end of therapy; (4) progression by RECIST after the end of therapy. Due to the relatively small number of patients in the clinical subgroups, only descriptive analyses were performed.

## 3 RESULTS

### 3.1 Population, treatment, and follow‐up

Thirty‐two cases from a pool of 70 sporadic DF patients treated with MTX‐VA at INT from May 1999 to June 2019 were identified. Patient's characteristics and treatment received are shown in Table 1. At baseline, 20 patients reported symptoms related to DF: pain was observed in 18 patients, functional impairment in 6 patients, other symptoms in 2 patients. Information about symptoms at baseline were lacking for 4 patients (12.5%), while 8 patients (25.0%) were asymptomatic at the start of chemotherapy. All patients had RECIST progressive disease (PD) at the time of chemotherapy start.

**TABLE 1:** Population characteristics of the overall cohort (*n* = 32 patients). MTX =methotrexate. RECIST =Response Evaluation Criteria In Solid Tumors

	*N* (%)
Gender	
females	26 (81.25%)
males	6 (18.75%)
Tumor site	
extremities/girdles	13 (40.63%)
abdominal wall	7 (21.88%)
thoracic wall	5 (15.62%)
neck	5 (15.62%)
intra‐abdominal	2 (6.25%)
Previous therapies	
no previous therapy	8 (25.00%)
surgery and radiotherapy	3 (9.38%)
surgery and chemotherapy	12 (37.50%)
surgery alone	4 (12.50%)
chemotherapy alone	5 (15.62%)
Presence of symptoms before therapy	
pain	18 (56.25%)
other (discomfort, bulk, functional impairment)	7 (21.88%)
no symptoms	8 (25.00%)
non assessable	4 (12.50%)
Administered chemotherapy	
MTX and vinblastine	25 (78.12%)
MTX and vinorelbine	7 (21.88%)
Reasons for therapy discontinuation	
treatment completion	30 (93.74%)
progressive disease according to RECIST	1 (3.13%)
therapy intolerance with stable disease	1 (3.13%)
Age (median, range)	40 years (14–68)
Therapy duration (median, range)	13.1 months (3.5–18.5)
	40 cycles (12–63)

Thirty patients (94%) completed the scheduled treatment, while 1 (3%) interrupted it for RECIST PD and 1 (3%) for therapy intolerance with stable disease (SD).

The median follow‐up duration was 47.6 months (range 11.4–234.0 months). After the therapy withdrawal, among the 20 initially symptomatic patients, 3 (15%) experienced worsening of symptoms, 14 (70%) remained clinically stable and for the other 3 (15%) no data were available. According to RECIST, 4 patients out of 32 (12.5%) had PD during follow‐up, 19 (59.4%) had SD and 7 (21.9%) had a partial response (PR), while data were missing for 2 (6.2%). Eight patients underwent further treatments: 2 (6.3%) had surgery and 6 (18.8%) had systemic therapy.

### 3.2 Response evaluation

**FIGURE 1:**
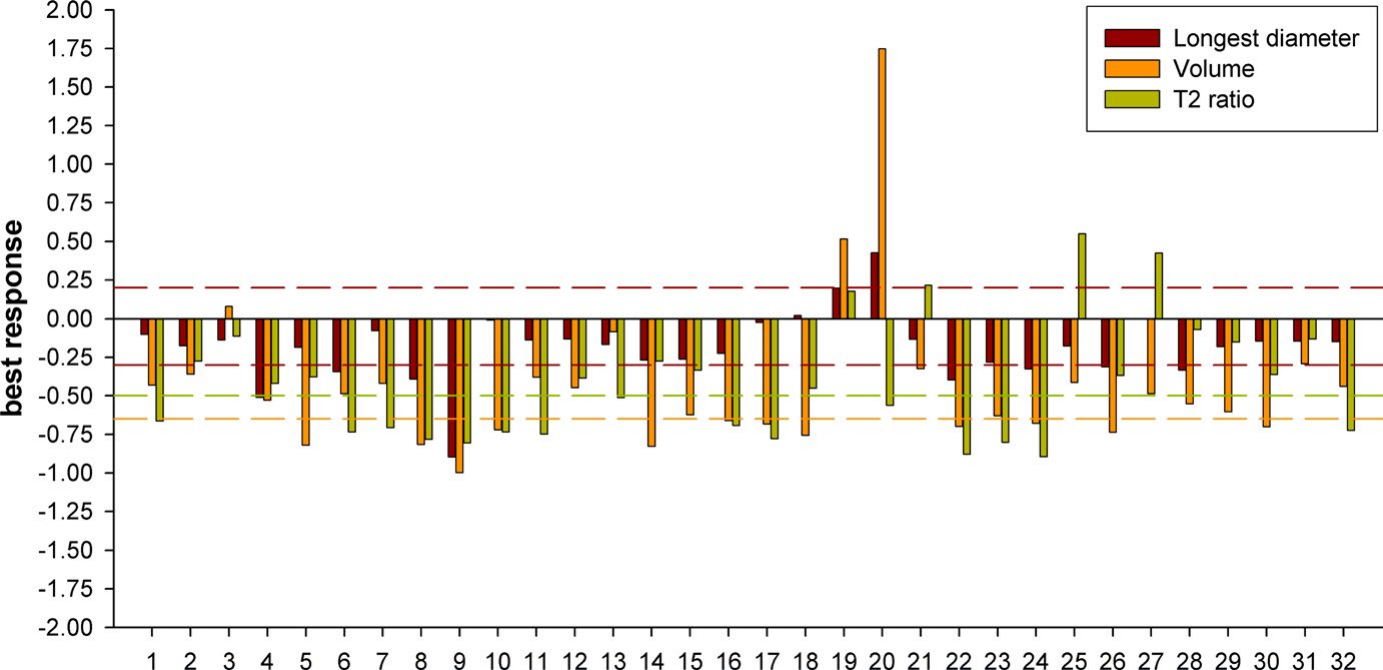
Bar plot with the *best response* according to the three radiological parameters on the y‐axis and the patients’ ID on the x‐axis. The red dashed lines at 0.20 and −0.30 indicate the Progressive Disease and Partial Response cut‐off of RECIST, respectively. The orange and green dashed line at −0.65 and −0.50 indicates the cut‐offs for volume and T2 intensity reduction, respectively

Figure 1 reports the best responses achieved during chemotherapy for each patient.

According to RECIST, the best response was: PR in 8 patients (25%), SD in 22 (69%; with a dimensional reduction in 20, no change in 1 and an increase in 1) and PD in 2 (6%). A tumor volume reduction of ≥65% was achieved in 12 subjects (38%), a <65% volume decrease in 17 (53%), and a tumor volume increase in 3 (9%). Seven patients (22%) had a small decrease or even an increase in longest diameter (formally a RECIST SD), while showing a tumor volume reduction ≥65% (lesions shrank without diminishing in the main diameter). A ≥50% decrease of normalized T2‐signal intensity was observed in 15 patients (47%), a <50% decrease in 13 (41%), and an increase in 4 (12%).

As reported in Figure 1, a general reduction of tumor maximum diameter, volume, and T2‐signal intensity was observed in the majority of patients. In three patients, a discrepancy between one‐dimensional/volumetric and T2‐signal intensity changes was observed. In particular, in two cases, the T2‐signal intensity increased despite a decrease in volume: one patient (ID27; volume −49%, T2‐signal intensity +43%) had no benefit during therapy, symptomatic worsening was observed during follow‐up and several further treatments were administered after chemotherapy withdrawal; another patient (ID25; volume −41%, T2‐signal intensity +55%) had no clinical benefit as well during therapy. Conversely, in one additional case (ID20), volume increased significantly during the first months of treatment (+175%), while T2‐signal intensity decreased (−56%), and the patient is asymptomatic after 4 years of follow‐up.

Among the 20 symptomatic patients (62.5%), symptomatic improvement during therapy was experienced in 15 (75.0%), while 4 (20.0%) had no symptomatic relief and 1 (5.0%) had symptomatic worsening. In particular, improvement of pain was observed in 13 patients, no improvement in 4 and worsening in 1. Improvement of functional impairment was found in five patients and no improvement in one.

### 3.3 Time trends of radiological parameters

**FIGURE 2:**
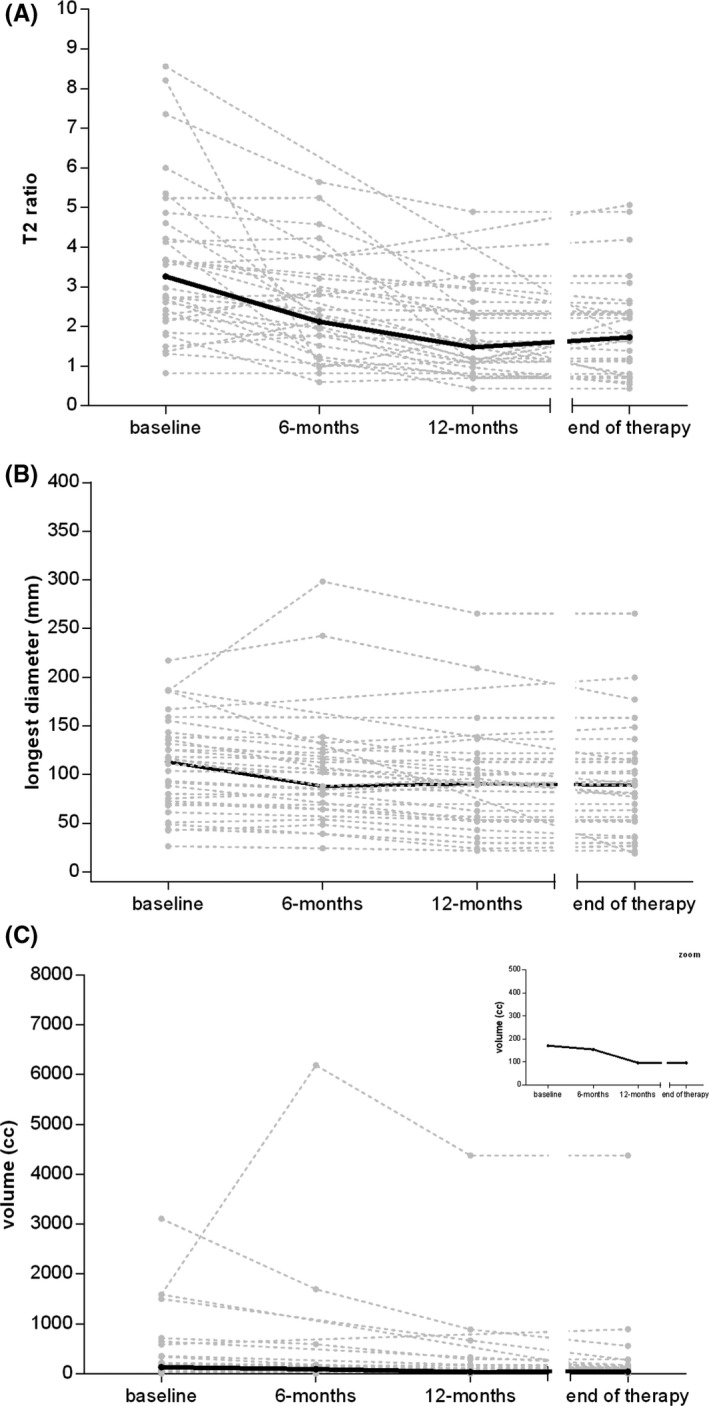
Spaghetti plots reporting the trend over time of (A) T2 ratio, (B) longest diameter, and (C) volume for each considered patient, respectively. A zoom window is also reported to aid the interpretation of the volume trend. Dots and dashed gray lines indicate the patients’ values and their trend over time. The solid black lines indicate the median values at each time point

The overall trend of the three radiological parameters over time is represented by spaghetti plots and descriptive statistics in Figure 2 and Table 2, respectively. The maximum diameter of the lesions showed small changes over time, with a median value of 112.9, 88.1, and 90.5 mm, respectively, at baseline, 6 and 12 months. Conversely, the volume of the lesions showed a reduction over time, with median values of 132.1, 91.1, and 37.4 cm^3^ among the time points; a reduction also in the interquartile range (i.e. IQR =75^th^ – 25^th^ centiles) values (302.0, 138.1, and 152.7 cm^3^, respectively) was observed. Similarly, we observed a gradual reduction over time of the normalized T2‐signal intensity of the lesions, both for median values (3.3, 2.1, and 1.5) and interquartile range (2.2, 2.0, and 1.3) at baseline, 6 and 12 months, respectively. Figure S1 reports the spaghetti plots drawn by considering as pivotal variables the relative change from baseline (i.e., percentage of baseline).

**TABLE 2:** Descriptive statistics of the trend over time of longest diameter (A), volume (B), and T2 ratio (C). IQR = Interquartile range

A. Longest diameter (mm)	*n*	min	25^th^ centile	median	75^th^ centile	max	IQR
baseline	32	26.40	71.60	112.95	140.70	217.30	69.10
6 months	23	24.20	64,10	88.10	122.50	298.30	58.40
12 months	27	21.70	53.10	90.50	115.90	265.60	62.80
end of therapy	32	19.00	53.20	89.05	114.80	265.60	61.60
**B. Volume (cm^3^)**							
baseline	32	2.900	47.835	132.100	349.840	3109.100	302.005
6 months	23	2.780	27.370	91.140	165.460	6188.700	138.090
12 months	27	1.700	17.100	37.400	169.800	4379.300	152.700
end of therapy	32	1.700	17.350	43.450	142.200	4379.300	124.850
**C. T2 ratio**							
baseline	32	0.825	2.249	3.264	4.412	8.560	2.163
6 months	23	0.598	1.236	2.120	3.212	5.640	1.976
12 months	27	0.437	0.966	1.474	2.312	4.890	1.347
end of therapy	32	0.437	0.802	1.728	2.353	5.070	1.551

IQR: 75^th^ centile‐ 25^th^ centile

### 3.4 Radiological parameters according to clinical variables

**FIGURE 3:**
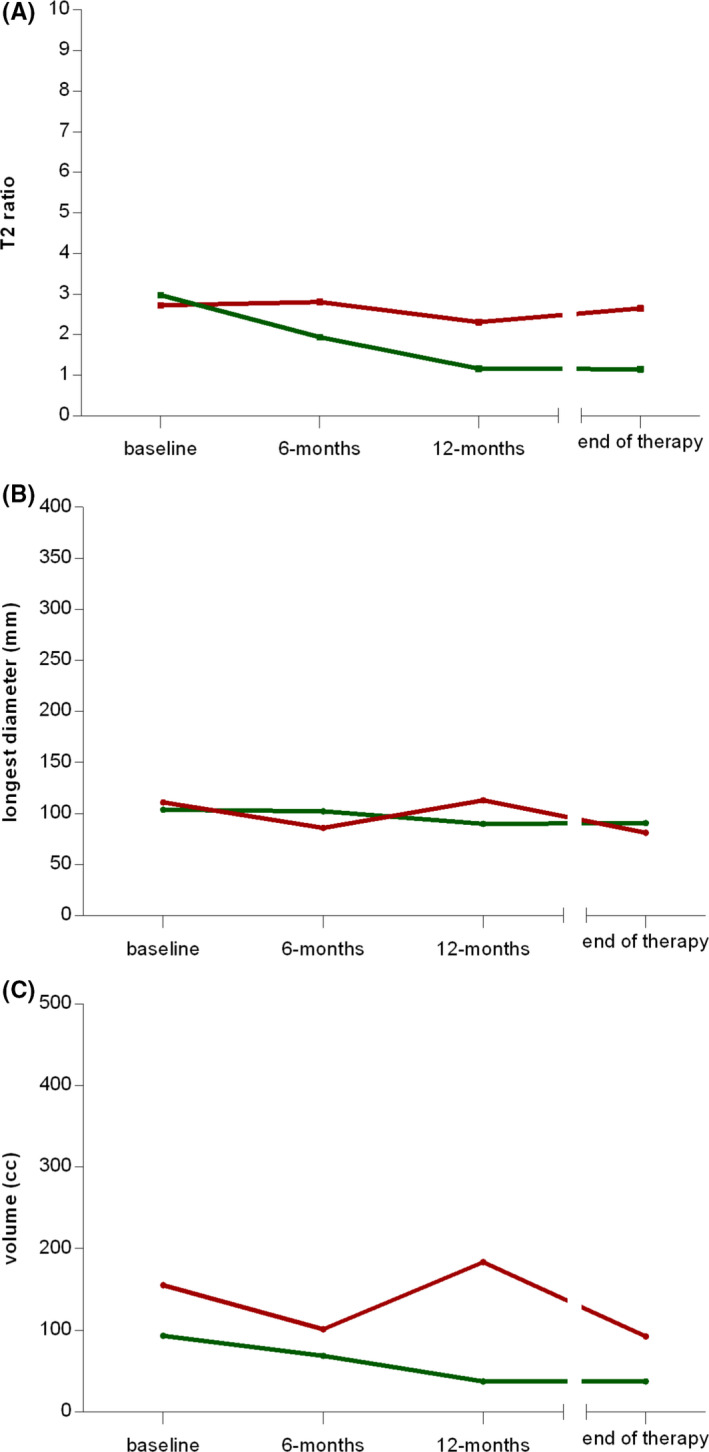
Spaghetti plots reporting the trend over time of (A) T2 ratio, (B) longest diameter, and (C) volume for each considered patient according to the *symptomatic improvement during therapy* (available for 20 patients). The red and green solid lines indicate the trend of the median values in the subgroups of patients without and with clinical improvement, respectively

In the subgroup of patients experiencing a symptomatic improvement during therapy (Figure 3), a reduction of the median T2‐signal intensity along the time points was observed (3.0, 1.9, 1.2, and 1.1 at baseline, 6 and 12 months and at therapy end, respectively), while in patients without symptomatic improvement a reduction was not observed (2.7, 2.8, 2.3, 2.6). The median maximum diameter and the median volume showed the following changes over time in patients with symptomatic improvement: 103.7, 102.3, 89.8, 90.7 mm and 93.4, 69.0, 37.4, 37.4 cm^3^, respectively; a clear trend was not deducible in patients without symptomatic improvement: 110.9, 85.8, 112.9, 81.1 mm and 155.4, 101.4, 183.4, 92.4 cm^3^, respectively.

**FIGURE 4:**
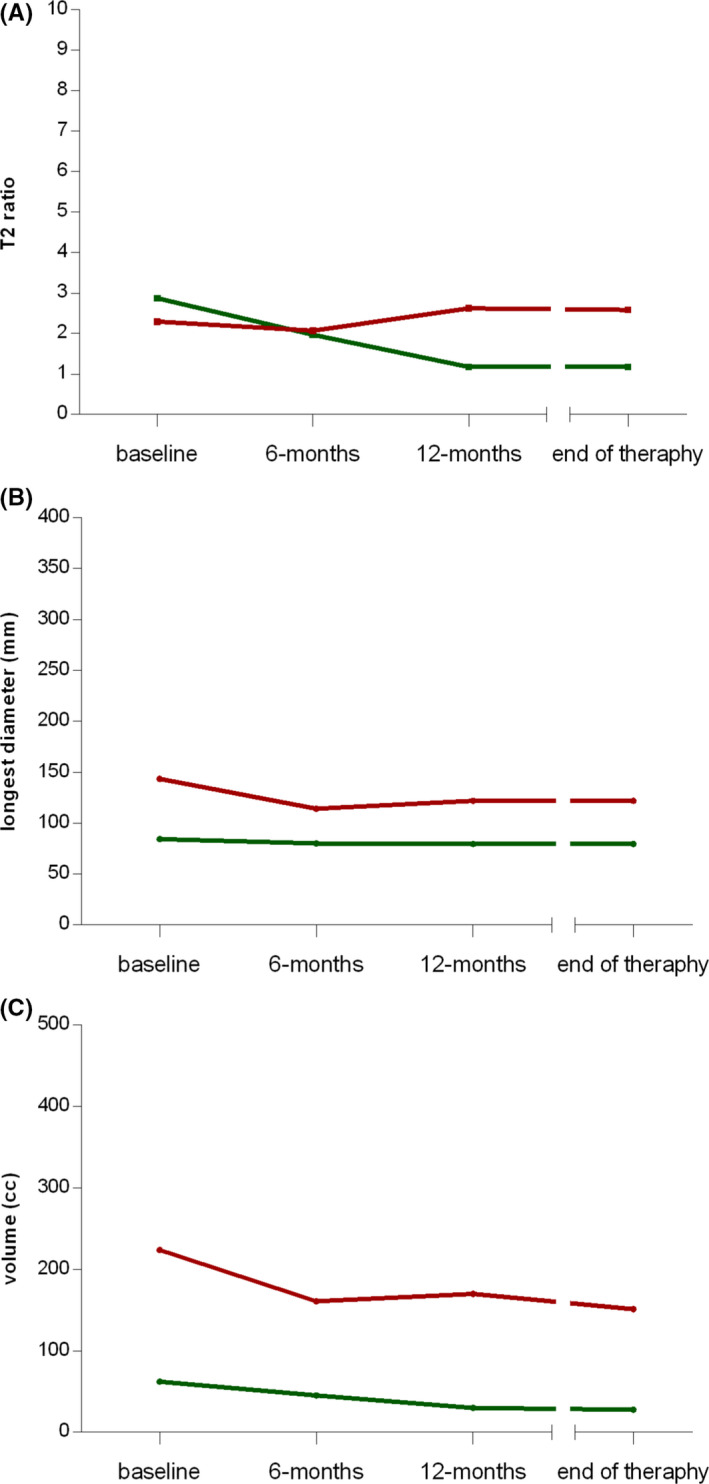
Spaghetti plots reporting the trend over time of (A) T2 ratio, (B) longest diameter, and (C) volume for each considered patient according to the *clinical progression during follow‐up* (available for 17 patients). The red and green solid lines indicate the trend of the median values in the subgroups of patients with and without clinical further progression, respectively

In the subgroup of patients maintaining the symptomatic improvement during follow‐up (Figure 4), a decrease of the median T2‐signal intensity during therapy was observed (2.9, 2.0, 1.2, 1.2), while in patients experiencing a clinical progression during follow‐up, a decrease was not found (2.3, 2.1, 2.6, 2.6). The median maximum diameter and the median volume had a similar trend in both subgroups: 84.2, 80.1, 79.4, 79.4 mm and 62.1, 45.2, 29.9, 27.6 cm^3^, respectively, in clinically non‐progressing patients; 143.4, 114.1, 121.9, 121.9 mm and 223.7, 160.9, 169.8, 151.2 cm^3^, respectively, in clinically progressing patients.

**FIGURE 5:**
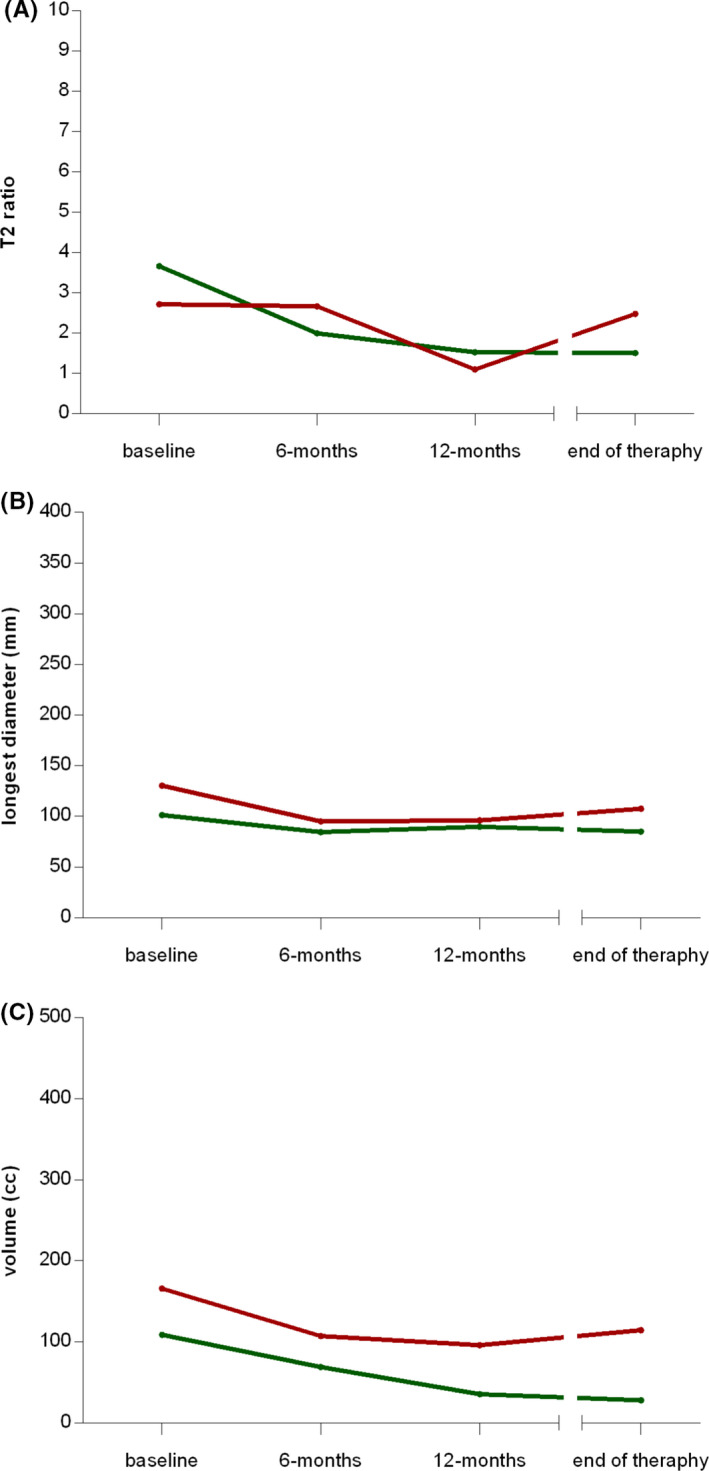
Spaghetti plots reporting the trend over time of (A) T2 ratio, (B) longest diameter, and (C) volume for each considered patient according to the *retreatment during follow‐up*. The red and green solid lines indicate the trend of the median values in the subgroups of patients with and without retreatment, respectively

As shown in Figure 5, a reduction of the median T2‐signal intensity was found in non‐retreated patients (3.7, 2.0, 1.5, 1.5), while an initial reduction and a subsequent increase at the end of treatment was observed in retreated patients (2.7, 2.7, 1.1, 2.5). The median maximum diameter and the median volume showed a similar trend in both subgroups: 101.4, 84.6, 89.8, 85.0 mm and 109.0, 69.0, 35.6, 28.1 cm^3^, respectively, in non‐retreated patients; 130.4, 95.2, 96.0, 107.8 mm and 165.8, 107.4, 96.0, 114.6 cm^3^, respectively, in retreated patients.

**FIGURE 6:**
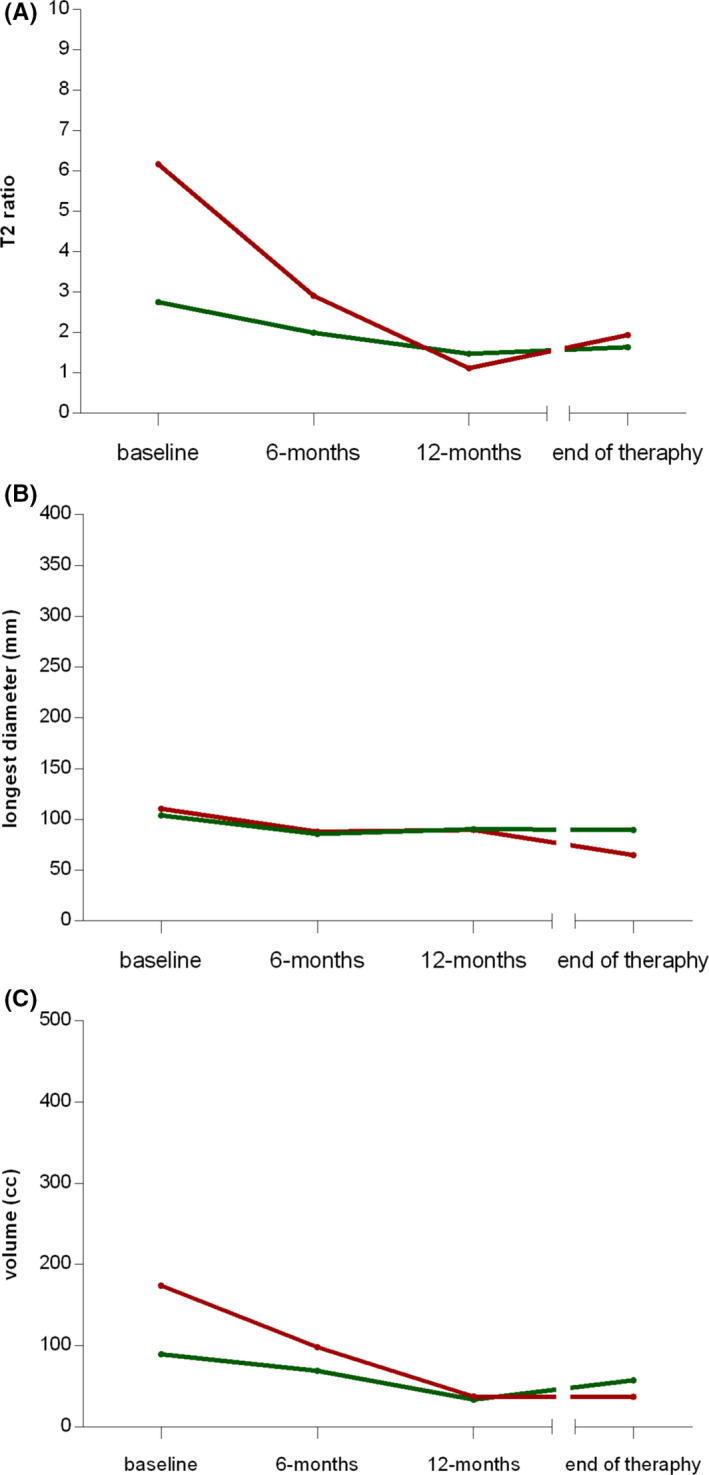
Spaghetti plots reporting the trend over time of (A) T2 ratio, (B) longest diameter, and (C) volume for each considered patient according to the *RECIST progression during follow‐up* (available for 30 patients). The red and green solid lines indicate the trend of the median values in the subgroups of patients with and without progression, respectively

Looking at progressions according to RECIST during follow‐up (Figure 6), the median T2‐signal intensity and the median volume were as following: 6.2, 2.9, 1.1, 1.9 and 174.1, 98.4, 37.4, 37.0 cm^3^, respectively, in progressing patients; 2.7, 2.0, 1.5, 1.6 and 89.5, 69.0, 33.8, 57.5 cm^3^, respectively, in non‐progressing patients. In the subgroup with RECIST progression, median volumes and T2‐signal intensity at baseline were higher.

Figures S2, –5 report the individual trends by considering the percentage of baseline according to the four clinical variables.

## 4 DISCUSSION

In this series of sporadic DF treated with low‐dose MTX‐VA, 25% of patients achieved a RECIST PR, while more responses were recorded based on volume and normalized T2‐signal intensity changes (38% and 47%, respectively). Few cases showed some discrepancies between the three radiological variables. When the radiological variables were evaluated according to the clinical variables, T2‐signal reduction seemed to be more informative about symptomatic improvement during therapy and future clinical progression.

This study has some limitations. First, a relatively small number of patients was included. Obviously, this reflects the rarity of this disease. Second, this was a retrospective analysis. Therefore, formal quality of life tests or tumor‐specific outcome tools was not used and there was a lack of standardization. Indeed, MRI examinations had been performed at different institutions (i.e., with different equipment and uneven parameters) and this may have caused some variations in measured T2‐signal intensity, even with a reference standard.^14^ Last, a unique reader analyzed images, with potential biases thereby. Nevertheless, measurements were performed using an objective method: we measured the signal intensity values, besides diameter and volume, using a slice‐by‐slice segmentation of the entire lesions and normalizing it to the normally appearing skeletal muscle. Indeed, contrary to CT, in which Hounsfield units represent an intrinsically standard metrics of tissue density, being standardized to attenuation coefficients of water and air, MRI T2‐signal is unitless and needs to be normalized to a reference point in order to be comparable between different examinations.^4^, ^20^ Other authors used different techniques, evaluating the signal intensity in a subjective way.^21^, ^22^, ^23^, ^24^

To our knowledge, however, this is the largest series of chemo‐treated patients with DF evaluated by MRI by using all three radiological criteria. In a population of 11 DF patients treated with sorafenib, Gounder et al^4^ described that changes in volume and T2‐signal intensity, rather than changes in tumor diameters, seemed to better reflect treatment effect. More recently, Shimizu et al^25^ studied 46 DF patients treated with meloxicam and showed that the low T2‐signal intensity area (measured at the maximal transverse section of the lesions) increased significantly during therapy in the group of non‐progressing patients. A Canadian group^21^ also reported its experience with low‐dose chemotherapy in patients with DF: among 22 patients with serial MRIs, they found a mean decrease in great diameter by 30%, a mean decrease in volume by 76%, and a decrease in T2 in 82% of the patients.

The rate of RECIST responses in the present series is superimposable to previously reported data.^11^, ^15^, ^21^, ^26^, ^27^, ^28^, ^29^, ^30^ However, RECIST seems to underestimate treatment response as compared to volumetric criteria: one‐dimensional responses were fewer than volumetric ones, even considering the linear‐volume equivalence when selecting the volumetric threshold.^19^ Most likely, this reflects the typical morphologic evolution of these tumors under systemic therapy, maintaining an elongated shape while shrinking and losing cellularity. Schiavon et al^19^ studied the use of volume as an alternative to one‐dimensional evaluation in imatinib‐treated GISTs, concluding that 3D changes may have the potential to be a more sensitive and precise marker of regression or progression, especially if the volume of the tumor was approximated to an ellipsoid.

By looking at the changes of the three radiological parameters altogether (Figure 1), we observed a similar pattern of reduction in the majority of patients. The most interesting and informative cases, however, were the few presenting discrepancies between the radiological variables, especially two patients who had an increase of T2‐signal intensity in spite of a reduction in maximal diameter and volume: both of them had no symptomatic improvement during therapy, suggesting a poor response^17^ and a more aggressive course of disease. Conversely, in the unique case showing no response in terms of volume (that actually increased substantially) but a relevant decrease of T2‐signal intensity during therapy, the patient is still asymptomatic after 4 years of follow‐up.

In the present analysis, an effort to investigate possible relationships between radiological variables and clinical ones has been made, taking into account that tumor response assessment through symptoms evaluation is crucial in this disease, where the quality of life improvement should be the goal of any treatment. A better knowledge of the radiological pattern of response, however, may be useful also to assess tumor response in asymptomatic patients, in which symptoms changes during treatment are not so informative. Interestingly, when we looked at the relation between radiological and clinical parameters, T2‐signal intensity proved to better reflect the symptomatic improvement during therapy and clinical progression during follow‐up. Similarly, Libertini et al,^31^ in a series of 32 DF patients treated with tamoxifen, found a lack of correlation between RECIST responses and clinical benefit during therapy; in their experience, however, also T2‐signal changes seemed not to correlate with clinical benefit. In the present series, no clear differences were apparent among retreated and non‐retreated patients during follow‐up as to any of the radiological measures: this was expected, because the decision to retreat or not is highly subjective, in the lack of any clearly established guidelines.

High volumes and high T2‐signal intensity values at baseline were shown to be a possible predictor of further RECIST‐progression during follow‐up. Kamali et al^23^ could not find any association between baseline MRI features of lesions and their behavior in a heterogeneous population of DF patients treated with several different systemic treatments. In the already mentioned study by Shimizu et al,^25^ the baseline MRI T2‐signal intensity was significantly higher among patients progressing on meloxicam as compared to those obtaining at least a stabilization.

In conclusion, we think that these results may well lead to validating a standardized MRI‐based algorithm as a tumor response indicator in a larger prospectively evaluated series of DF patients treated with systemic therapy, with the support of disease‐specific quality of life tests.

## CONFLICTS OF INTEREST

All Authors have no potential conflicts of interest to declare.

## AUTHOR CONTRIBUTIONS

Edoardo Zanchetta, Elena Palassini, Paolo Giovanni Casali, Chiara Maura Ciniselli, Paolo Verderio, Carlo Morosi, Alessandra Casale and Francesca Gabriella Greco: conceptualization and methodology. Edoardo Zanchetta, Elena Palassini, Silvia Stacchiotti, Giacomo Giulio Baldi, Salvatore Provenzano, Rossella Bertulli, Federica Bini, Alessandra Casale, Francesca Gabriella Greco, Andrea Ferrari, Marco Fiore, Alessandro Gronchi, Chiara Colombo, Paolo Giovanni Casali: investigation. Edoardo Zanchetta, Chiara Maura Ciniselli and Annalisa Bardelli: formal analysis and visualization. Paolo Giovanni Casali, Paolo Verderio and Carlo Morosi: supervision. Edoardo Zanchetta and Elena Palassini: writing‐original draft. All authors: writing review and editing.

## ETHICAL STATEMENT

Institutional ethics committee's approval was obtained.

## Supporting information

Fig S1

Fig S2

Fig S3

Fig S4

Fig S5
